# Gut Microbiota and Their Metabolites: The Hidden Driver of Diabetic Nephropathy? Unveiling Gut Microbe's Role in DN

**DOI:** 10.1111/1753-0407.70068

**Published:** 2025-04-06

**Authors:** Jinzhou Liu, Min Guo, Xiaobin Yuan, Xiao Fan, Jin Wang, Xiangying Jiao

**Affiliations:** ^1^ Department of Physiology The Key Laboratory of Physiology of Shanxi Province, the Key Laboratory of Cellular Physiology of Ministry of Education, Shanxi Medical University Taiyuan China; ^2^ Department of Urology First Hospital of Shanxi Medical University Taiyuan China

**Keywords:** diabetic nephropathy, gut microbiota, gut–kidney axis, microbial metabolites, novel biomarkers

## Abstract

**Background:**

Diabetic nephropathy (DN) is a severe microvascular complication of diabetes with a complex pathogenesis.

**Methods:**

Recent studies were reviewed to explore the role of gut microbiota and its metabolites in DN development.

**Results:**

Dysbiosis of gut bacteria contributes to pathological changes such as glomerular sclerosis and renal tubule injury. Microbial metabolites are involved in DN through immune and inflammatory pathways.

**Conclusions:**

Understanding the relationship between gut microbiota, its metabolites, and DN may offer potential implications for DN diagnosis, prevention, and treatment. Translating this knowledge into clinical practice presents challenges and opportunities.

## Graphical abstract

Gut microbiota influences the progression of diabetic nephropathy through gut‐kidney axis.

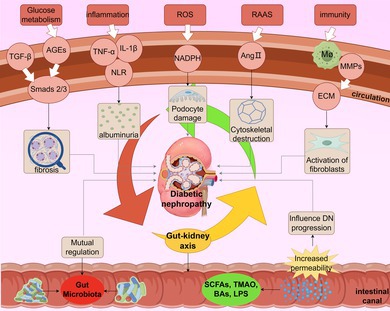

**Summary**

- The disordered genera and species of intestinal microbe and their metabolites in diabetic nephropathy reported in recent years are summarized.
- The latest strategy and treatment based on gut microbiota for preventing and treating diabetic nephropathy are summarized and ingeminated.
- The crucial role of gut microbiota and its metabolites in diabetic nephropathy is illustrated, providing new ideas for DN treatment and alleviating the further deterioration of renal function.

## 1 Introduction

The latest data from International Diabetes Federation (IDF) point to around 537 million adults aged 20–79 are living with diabetes worldwide, equivalent to one in 10 people, and predict that the number will increase 46% over 783 million by 2045 [1]. Worse still, the IDF's report on “Diabetes and Kidney Disease” released in 2023 pointed out that the incidence of chronic kidney disease caused by type 2 diabetes has increased by 74% globally in the past 20 years, with over 147 million people affected by DN coming from China [2]. The incidence of DN is high, the progression of the disease is hidden, and the decades‐long treatment process brings great economic burden to patients, which makes their quality of life decline, and eventually leading to end‐stage renal disease (ESRD). Furthermore, it stands as the primary cause of mortality among individuals afflicted with both type 1 and type 2 diabetes [3, 4, 5, 6]. In fact, the definition of DN is relatively vague, and it is difficult to accurately define DN in epidemiology or clinical practice. It is generally believed that the clinical manifestations of DN are proteinuria, podocyte dedifferentiation, epithelial‐mesenchymal transformation, and elevated blood pressure [7]. Pathologic changes seen in DN are interstitial inflammation, thickening of the tubular basement membrane, tubular atrophy, and interstitial fibrosis [8]. Current studies have found that the pathogenesis of DN can be roughly divided into glucose metabolism disorder, inflammation, oxidative stress and RAAS system activation.

Gut microbiota has been the focus of medical research in recent years, and it plays an important role in physiology and disease states, including obesity, diabetes, asthma, irritable bowel syndrome, cancer, cardiovascular disease, and aging [9, 10, 11]. Recent studies have shown that the imbalance of gut microbiota may be related to the occurrence and development of a variety of diseases, including DN [12, 13, 14, 15, 16]. Among various microbiota phyla in healthy adults, Firmicutes and Bacteroidetes dominate, accounting for about 90% [17]. In the pathological state, the original relatively stable balance of intestinal microorganisms is destroyed, which may lead to endotoxins and pathogens passing through the intestinal barrier, destroying the protective effect of intestinal mucosa, allowing harmful substances that should be confined in the intestinal cavity to enter the blood, triggering a systemic inflammatory response, which is an important factor in the occurrence of diabetes and its complications [18]. Gut microbiota of certain metabolic products, such as short chain fatty acids (SCFAs), has been shown to maintain intestinal health and regulate balance and plays an important role in the host. SCFAs may not only improve insulin sensitivity but also reduce inflammatory responses, thus potentially having a positive impact on the prevention and treatment of DN. In addition, the gut microbiota also probably by regulating blood pressure and oxidative stress levels, directly affects the health of the kidney [19].

**FIGURE 1:**
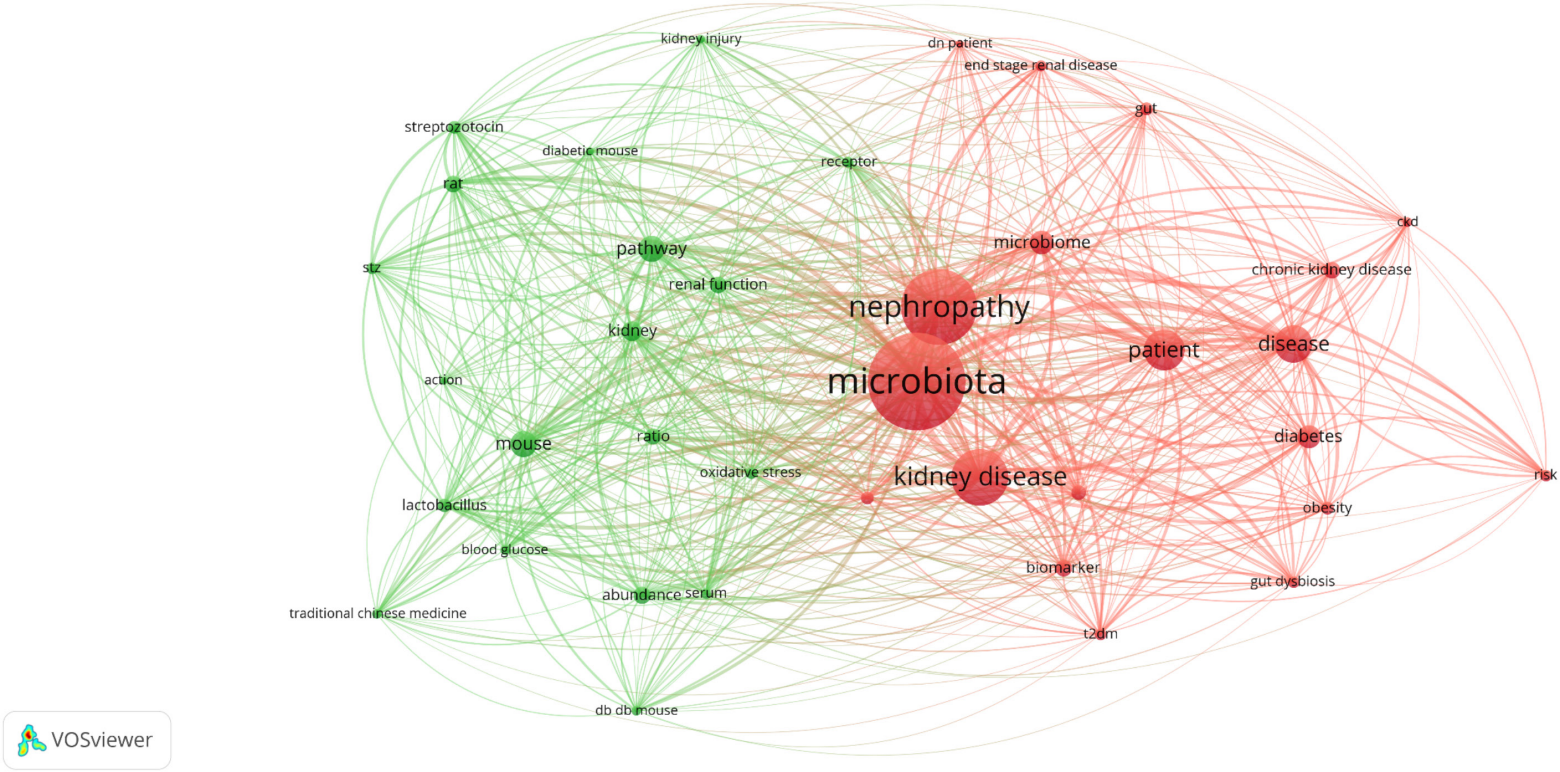
Visualization of keyword networks by using VOSviewer.

After conducting an analysis of 151 papers with keywords such as “gut microbiota” and “DN” over the past 5 years, all relevant records were extracted and imported into VOSviewer (version 1.6.16) for bibliometric analysis. The findings are presented in Figure 1. The research primarily focuses on the relationship between gut microbiota, kidney disease, and biomarkers, suggesting a potential link between the dysbiosis of gut microbiota and the occurrence and progression of DN. Here, we review recent clinical trials, animal studies, and multiple potential biomarkers including protein biomarkers, proteomics, metabolomics, and transcriptomics to summarize current research on the role of gut microbiota and microbial metabolites in DN progression (Tables 1 and 2).

**TABLE 1:** Changes in the composition of intestinal microorganisms in DN.

Phylum	Subjects	Altered genus	Altered species	References
Firmicutes	Human	**↑:** Anaerotruncus, Acidaminococcus, Catenibacterium, Clostridium, Coprococcus, Coprococcus_1, Christensenella, Christensenellaceae_R_7_group, Clostridium_innocuum_group, Eubacterium, Eisenbergiella, Flavonifractor, Lactococcus, Lachnoclostridium, Lactobacillus, Marvinbryantia, Megasphaera, Mitsuokella, Subdoligranulum, Turicibacter	Clostridium_sensu stricto 1, Clostridium‐XIVa, Clostridium‐XVIII, *Eubacterium hallii* , *Megasphaera elsdenii* , *Ruminococcus torques* , *Ruminococcus gnavus* group	[20, 21, 22, 23, 24, 25, 26, 27, 28, 29]
		**↓:** Anaerostipes, Coprococcus2, *Eubacterium ventriosum* group, *Eubacterium coprostanoligenes* group, *Eubacterium xylanophilum* group, Lactococcus, Lachnoclostridium, Lactobacillus, Phascolarctobacterium, Roseburia	Clostridium sp. CAG_768, Clostridium sp. 26_22, Eubacterium sp. AF22_9, *Ruminococcus gauvreauii* group, *Roseburia intestinalis* , Roseburia sp. AM23_20, Tyzzerella_3	[20, 24, 25, 26, 27, 30]
	Rats	**↑:** Acetanaerobacterium, Anaerotruncus, Clostridium, Enterococcus, Erysipelatoclostridium, Erysipelotrichaceae_UCG‐003, Faecalibacterium, Hungatella, Kurthia, Lactobacillus, Lachnospiraceae_NK4A136_group, Negativibacillus, Ruminococcus_1, Streptococcus, Subdoligranulum, Turicibacter	Eubacterium_siraeum_group, Eubacterium_coprostanoligenes, Ruminococcaceae_UCG‐014, Ruminococcus_1	[31, 32, 33, 34, 35, 36, 37, 38]
		**↓:** Anaerotruncus, Allobaculum, Anaerovibrio, Butyricicoccus, Blautia, Clostridia_UCG‐014, Dubosiella, Eubacterium. Faecalibacterium, Fusicatenibacter, Lactobacillus, Ligilactobacillus, Lachnospiraceae_NK4A136_group, Roseburia	Ruminococcaceae UCG‐005	[18, 31, 35, 36, 37, 38, 39, 40]
Bacteroidota	Human	**↑:** Alistipes, Butyricimonas, Barnesiella, Bacteroides, Prevotella, Prevotella_7	Alistipes ihumii, *Bacteroides plebeius* , *Bacteroides stercoris* , *Bacteroides stercoris* CAG_120, Prevotella sp. MSX73, Tannerella sp. CAG_51	[20, 23, 25, 27, 28, 29, 30]
		**↓:** Muribaculum.	*Bacteroides coprocola* , *Bacteroides xylanisolvens* , Bacteroides sp. D22, *Bacteroides plebeius* CAG_211, Prevotella_9	[25, 29, 30]
	Rats	**↑:** Alloprevotella, Bacteroides, Paraprevotella, Prevotellaceae UCG_001, Streptococcus, Rikenella.		[31, 32, 33, 41]
		**↓:** Alistipes, Alloprevotella, Bacteroides, Muribaculum, Odoribacter, Parabacteroides, Rikenella.		[18, 31, 32, 37, 38, 42]
Pseudomonadota	Human	**↑:** Helicobacter, Klebsiella, Parasutterella, Sutterella	*Bilophila wadsworthia* , *Escherichia coli* , Escherichia‐Shigella	[22, 24, 25, 27, 29, 43]
	Rats	**↑:** Acinetobacter, Anaerobiospirillum, Citrobacter, Desulfovibrio, Klebsiella	Escherichia‐Shigella	[18, 31, 32, 34, 35, 36, 37, 41, 42]
Proteobacteria	Human	**↑:** Parasutterella, Helicobacter		[25, 27]
	Rats	**↑:** Neisseria		[31]
Verrucomicrobiot	Human, rats	**↑:** Akkermansia		[20, 22, 26, 27, 44]
Actinomycetota	Human	**↑:** Olsenella		[24]
	Rats	**↑:** Bifidobacterium		[36, 38]
Fusobacteriota	Human	**↑:** Fusobacterium		[22]
	Rats		**↑:** *Fusobacterium varium*	[30]
Ascomycota	Rats	**↑:** Candidatus_Saccharimonas		[18]
Deferribacterota	Rats	**↓:** Mucispirillum		[31]
Spirochaetota	Rats	**↑:** Treponema		[18]

**TABLE 2:** Microbial metabolites, their producers, and their role in the pathogenesis of DN.

Metabolite	Microbial species (Genus)	Role of metabolite in DN pathogenesis
Short‐chain fatty acids	Faecalibacterium, Ruminococcus, Clostridium, Bifidobacterium	Enhance gut barrier function, reduce inflammation and oxidative stress, potentially delaying DN progression [21, 22, 27, 45, 46]
Bile acids (BAs)	Bacteroides, Lactobacillus, Bifidobacterium	Influence lipid metabolism and inflammation; may regulate kidney fibrosis and progression of DN through FXR/GPR receptor pathways [21, 27, 47]
Lipopolysaccharide	Escherichia	Induce systemic inflammation through TLR4/NF‐κB pathway, aggravating DN‐related inflammation and injury [42, 44]
Branched‐chain amino acids (BCAAs)	Prevotella	BCAAs are associated with insulin resistance, potentially exacerbating DN and kidney dysfunction [48, 49]
Indole, indoxyl sulfate	Bacteroides, Klebsiella	Activate aryl hydrocarbon receptor (AhR), contributing to renal inflammation and fibrosis, worsening DN progression [47, 50]
Lactic acid	Lactobacillus	Exhibits anti‐inflammatory effects, potentially improving DN by stabilizing gut microbiota and modulating immune responses [42, 51]
Uremic toxins	Enterococcus	Induce systemic inflammation and renal fibrosis, exacerbating DN pathology [38, 52]

## 2 Intestinal Changes in Diabetic Nephropathy

### 2.1 Changes in the Intestinal Barrier

The host is protected from harmful toxins and pathogens in the environment by the intestinal barrier [53]. Pathologically, oxidative stress, hypoxia, and excessive bacterial presence can increase the permeability of the intestinal barrier, leading to abnormal pathological phenomena [54]. The occurrence and progression of kidney diseases are often accompanied by the imbalance of gut microbiota, which results in diarrhea and other symptoms [55]. When the intestinal barrier is destroyed, harmful substances would enter the systemic circulation ectopically in DN patients [56, 57]. Recent studies revealed DN is associated with the loss of apical junction complex, specifically occludin and claudin‐1; this may be caused by reduced renal filtration in DN patients and increased ammonia reabsorption due to large amounts of urea hydrolysis [58, 59].

Urea enters the intestinal lumen from systemic circulation and hydrolyzes into alkaline ammonium hydroxide, which might aggravate intestinal mucosal injury [60]. Lipopolysaccharide (LPS), present in the majority of microorganisms cell walls, plays a crucial role in intestinal barrier breakdown [61]. Recently, Kajiwara et al. proposed that TLR2/4 could induce nephropathy in diabetic mice through *Porphyromonas gingivalis* lipopolysaccharide (Pg‐LPS) [62]. In addition, Wada et al. and Kim et al. have shown that blocking TLR2/TLR4‐NLRP3 pathway activation reduces urinary albumin excretion and improves diabetes [63, 64]. Moreover, Indoxyl, *p*‐cresyl sulfate, and cresol may also contribute to the systemic exacerbation of DN due to disruption of the intestinal barrier [65, 66].

The function of the intestinal barrier is not limited to physical isolation, but also includes multiple functions such as immune regulation and nutrient absorption [67]. Studies have found that damage to the intestinal barrier may lead to further deterioration of kidney function, and this bidirectional regulatory mechanism emphasizes the interaction between the gut and the kidney [55]. In addition, damage to the intestinal barrier may also trigger systemic inflammation, further increasing the burden on the kidney. For example, acute pancreatitis can lead to damage to the intestinal barrier, leading to multiple organ dysfunction [68]. Therefore, protection of the intestinal barrier not only contributes to intestinal health, but also has a positive effect on kidney function recovery.

### 2.2 The Gut‐Kidney Axis in DN

The concept of the gut‐kidney axis is believed to have been initially proposed by Ritz at the 2011 Dialysis Congress. This proposal was based on the discovery that the level of endotoxin translocated from the gut to the bloodstream in hemodialysis patients is linked to frequent episodes of hypotension and cardiac paralysis during dialysis [69]. Ritz termed this phenomenon “enterorenal syndrome.” Additionally, it has been suggested that Meijers and Evenepoel further refined the concept [70], associating it with the progression of end‐stage renal disease (ESRD) and chronic kidney disease (CKD). In the same year, Stef et al. proposed that this theory could be applied to treat conditions associated with elevated calcium oxalate (CaOx) levels.

The gut‐kidney axis is a complex and delicate system, in which the gut microbiota interact with the kidney, and the pathological state will change accordingly. Dysregulation of gut microbiota is recently considered to be one of the important factors contributing to kidney disease, particularly in the context of acute kidney injury (AKI) and CKD [71]. The diversity and community intestinal microbiota in various categories are intricately associated with the development of DN. The intestinal microbiota of patients with DN has undergone significant changes, among which the changes in the number and types of microorganisms, such as *Escherichia coli* , Bifidobacteria, Lactobacillus, yeast, and Eubacteria, are related to the increased risk of DN [42, 47, 72]. With the development of DN, the diversity of intestinal microbiota in patients will also begin to be imbalanced [20]. In addition, studies have demonstrated that specific microbial metabolites can influence the progression of kidney disease by affecting renal hemodynamics and inflammation [73]. Moreover, there is a difference between type 1 and type 2 diabetes, in which the bacteria have a specific relationship with DN [44]. Tao et al. demonstrated the presence of the gut‐kidney axis in 42 patients, and could analyze whether patients developed DN through identification of gut microbiota [43]. Many studies have confirmed that the gut‐kidney axis is a bidirectional process; this interaction may affect kidney function and response to injury [74]. For example, changes in the gut microbiota can lead to increased kidney inflammation, thus affecting the prognosis of the kidney [75]. In addition, uremic toxins produced by the gut microbiota, such as trimethylamine N‐oxide (TMAO) and advanced glycation end products (AGEs), are strongly associated with the progression of kidney disease [76]. The accumulation of these toxins not only aggravates the kidney injury, but also may form a vicious cycle by affecting the composition of intestinal microbiota [77]. SCFAs are the main energy source of intestinal epithelium. Intestinal ecological disorder can reduce the production of SCFAs, increase the fermentation of proteolytic bacteria, produce urinary toxins, and exert their nephrotoxic effect [78, 79]. In contrast, amino acid end products are converted to urea in the liver, which is excreted from the colon due to decreased renal clearance. This will induce the multiplication of urea bacteria and aggravate the intestinal imbalance [80, 81]. Most studies on the gut‐kidney axis have focused on drugs; probiotics, traditional Chinese medicines, and biological extracts have shown the ability to reduce DN via the gut‐kidney axis [41, 82, 83, 84, 85, 86]. Future research on the mechanism and more details is expected to reveal the mystery of the gut‐kidney axis more accurately and intensively.

### 2.3 Changes of Gut Microbiota in DN

Changes in intestinal permeability and the gut‐kidney axis result in dysregulation of gut microbiota. In fact, changes in the gut microbiota may also be related to factors such as diet, lifestyle, and drug use in people with diabetes. By eliminating the influence of interfering factors as much as possible, and by analyzing the structural changes of gut microbes, we can better guide diabetic patients to make diet and lifestyle adjustments, as well as rational choices of drugs. In the latest study, both Lu and Zhang et al. observed differences in gut microbiota between diabetic patients (DM) and DN patients without kidney damage. At the genus level, there was a significant increase in the abundance of *Romboutsia*, *Faecalibacterium*, *Acidaminococcus*, *Megasphaera*, and *Sutterella* in the DM group. In contrast, the abundance of *Christensenella*, *Clostridium‐XIVa*, *Eisenbergiella*, *Fusobacterium*, *Parabacteroides*, *Ruminococcus_gnavus*, *flavone factors*, and *Clostridium‐XVIII* in the DN group was significantly increased [21, 22]. Thus, the changes of DN and microbiota are inseparable, leading to alteration in the gut environment that affects gut barrier function and immune response.

We investigated 151 studies over the past 5 years and systematically collated and summarized changes in the composition of intestinal microorganisms in DN based on human and rat research (Table 1). At the generic level, the up‐regulated bacteria in DN are *Akkermansia*, *Bacteroides*, *Clostridium*, *Escherichia‐Shigella*, *Megasphaera*, and *Ruminococcaceae*, etc. [23, 24, 31, 32, 33, 34, 39, 43]. However, the down‐regulated bacterial groups in DN at the genus level included *Anaerostipes*, *Clostridium* sp. *CAG_768*, *Muribaculaceae*, *Roseburia*, etc. [24, 25, 31, 35]. This indicates that there are significant differences in the structural characteristics of the gut microbiota in DN patients compared with healthy people. Such changes may affect intestinal barrier function and immune responses. In the context of gut microbiota presenting us with myriad possibilities, it is imperative to acknowledge that current research in this area remains at a nascent stage, characterized by numerous influencing variables and potential contradictory findings upon investigation. There is some debate about the variation in the microbiota of *Actinobacteria*, *Akkermansia*, *Lactobacillus*, *Lachnoclostridium* and *Ruminococcaceae* [24, 26, 32, 35, 47]. The upregulation and downregulation findings are inconsistent, which we infer is due to the fluctuation in species and diet. In view of the changes in gut microbiota, we believe that researchers can then use antibiotics to intervene in the structure of gut microbiota and observe the changes in metabolism and immune function of DN patients.

### 2.4 The Role of Microbial Metabolites in DN

#### 2.4.1 Short‐Chain Fatty Acid

The intestinal microbiota metabolite short‐chain fatty acids (SCFAs) can be used as energy sources absorbed through the colon mucosa [87, 88, 89]. In DN, SCFAs function after synthesis by activating transmembrane G‐protein‐coupled receptors (GPCRS) of GPR41 and GPR43 or inhibiting histone deacetylation (HDAC) directly in host cells [90]. Meanwhile, dietary fiber has been shown to prevent DN by activating GPR43 and GPR109A through SCFAs [91]. In the study by Zhou et al., butyrate‐induced histone lysine butyration was found to prevent proteinuria and renal failure, as well as inhibit renal inflammation and fibrosis [92]. It has been found that exogenous supplementation of SCFAs and monocyte chemotactic protein‐1 (MCP‐1) can inhibit the expansion of mesangial cell lines, the production of reactive oxygen species (ROS) and the expression of pro‐inflammatory cytokines, thereby improving renal fibrosis [93]. On the other hand, by inhibiting GPR43‐mediated NF‐κB signaling and ROS, butyrate supplementation significantly improved hyperglycemia, improved renal function, and inhibited renal fibrosis in DN mice induced by high‐fat diet (HFD) and streptozotocin (STZ) [51]. Therefore, it is particularly important to further study the specific mechanism of action of SCFAs in the kidney.

#### 2.4.2 Trimethylamine‐N‐Oxide

TMAO is a small organic compound that has gained significant attention in recent years due to its role as a risk factor for various chronic diseases, such as CKD, type 2 diabetes, and cancer [94, 95]. TMAO originates from the metabolism of dietary nutrients, including choline, carnitine, and betaine, which are converted into trimethylamine (TMA) by gut microbiota [96]. TMA is then absorbed into the bloodstream and transported to the liver, where it is oxidized into TMAO by flavin monooxygenases (FMOs) [97]. This process highlights the critical role of both diet and gut microbiota in TMAO generation.

TMA is an important intestinal metabolite, and in vitro studies have identified six microbial groups of 79 strains associated with TMA/TMAO production, including *Anaerococcus hydrogenalis* DSM 7454, *Clostridium hathewayi* DSM 13749, *Edwardsiella tarda* ATCC 23685, and *Proteus penneri* ATCC 35198 [94, 98]. High levels of TMAO are linked to altered gut microbiota composition, such as an increase in Firmicutes relative to Bacteroides and lower overall microbial diversity [97].

The kidneys control the clearance of TMAO under normal physiological conditions [99, 100]. TMAO‐mediated inflammation has become recognized as a significant risk factor for CKD progression. For example, Zhang et al. demonstrated that high TMAO concentrations promote vascular calcification in CKD rats by activating the NLRP3 inflammasome and NF‐κB signaling pathways [99]. Emerging evidence suggests that TMAO is closely linked to the progression of DN. Yu et al. found that a disturbed gut microbiota, increased TMAO levels, and decreased eGFR form a negative feedback loop, leading to renal function deterioration [101]. Furthermore, TMAO's involvement in inflammation and its inverse relationship with eGFR make it a potential biomarker for detecting DN at an earlier stage [102]. This biomarker role could improve diagnostic accuracy and help guide future studies aiming to delay DN progression through targeted therapies.

While TMAO has well‐documented pro‐atherogenic and pro‐thrombotic properties, its role in DN may involve alterations in macrophages and platelets, both of which are key players in the pathophysiology of DN [103, 104]. Macrophages are central to the inflammatory responses in DN, contributing to both chronic inflammation and fibrosis in the kidney. Studies have shown that TMAO can activate inflammatory signaling pathways, such as NF‐κB and NLRP3 inflammasome, which may indirectly promote macrophage activation in the renal microenvironment [105]. This activation could exacerbate renal damage through the release of pro‐inflammatory cytokines and profibrotic factors [106].

In addition, TMAO's pro‐thrombotic effects may influence platelet activity, which is often dysregulated in diabetes [107]. Platelet hyperactivation is known to contribute to microvascular complications in DN by promoting glomerular injury and microthrombosis [108]. While the direct role of TMAO in platelet‐mediated damage in DN has not been fully elucidated, its established effects on platelet reactivity and clot formation suggest that it could exacerbate microvascular injury in the diabetic kidney [107]. Future studies are needed to further explore these mechanisms and clarify their relevance to DN progression.

#### 2.4.3 Bile Acids

Bile acids (BAs) refer to a large group of cholic acids that exist in the form of sodium or potassium salts in bile [109]. Metagenomic analysis revealed that Firmicutes, Bacteroides, Lactobacillus, Bifidobacterium, and Clostridium play a crucial role in the formation of secondary BAs [45, 46]. In BAs, ursodeoxycholic acid (UDCA) is formed by the 7α/β isomerization of chenodeoxycholic acid (CDCA), which can be induced by Clostridium absonum [110]. Hydroxyl groups on the 3, 7, or 12 rings are oxidized to produce BAs by bacteria with hydroxyl steroid dehydrogenase (HSDs), an enzyme found in the phyla Actinomyces, Proteobacteria, Firmicutes, and Bacteroidetes [111].

Studies have shown that DN is associated with significant alterations in bile acid profiles. In DN patients, secondary bile acids such as lithocholic acid (LCA) and deoxycholic acid (DCA) are significantly elevated, whereas primary bile acids such as chenodeoxycholic acid (CDCA) and cholic acid (CA) may be reduced [112]. These alterations suggest gut microbiota dysbiosis and altered bile acid metabolism in DN. Furthermore, different bile acids exhibit differential effects on farlactone X receptor (FXR) and Takeda G protein‐coupled Receptor 5 (TGR5) [113]. CDCA is the most potent agonist for FXR, while LCA and DCA are known to strongly activate TGR5 [114]. In contrast, UDCA has relatively weaker effects on both receptors but may still exert beneficial effects through other mechanisms, such as anti‐inflammatory properties [115]. BAs can activate several nuclear hormone receptors, notably FXR and TGR5 [116]. FXR is a transcription factor that binds to promoter regions and initiates the expression of multiple target genes [45]. BAs synthesis was inhibited by FXR negative feedback [117]. Treatment of diabetic DBA/2 J and db/db mice with FXR/TGR5 bisagonist Int‐767 has been found to improve proteinuria and prevent podocyte injury, mesangial dilation, and tubulointerstitial fibrosis [116]. TGR5 is another bile acid response receptor that is involved in host metabolism [118]. Recent studies indicate that LCA and DCA are potent TGR5 activators, whereas CDCA strongly activates FXR [113]. These interactions may explain the differential roles of bile acids in DN.

The microbiome has the ability to modify bile acid receptors FXR and TGR5, and targeting their interactions may offer a potential pathway for treating DN.

#### 2.4.4 Lipopolysaccharide

**FIGURE 2:**
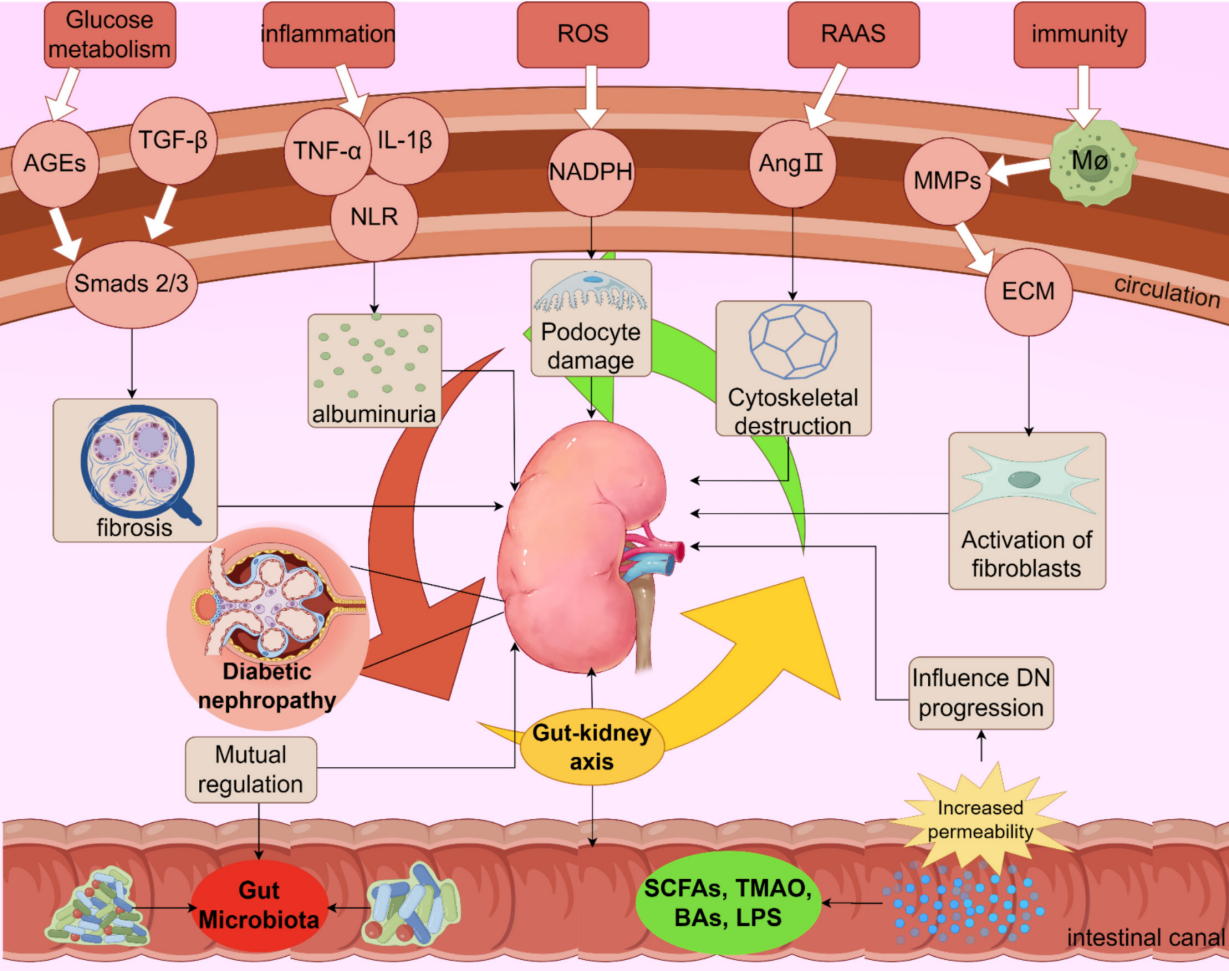
Association of gut microbiota to influence DN development.

LPS plays an important role in host‐microbial interactions [119]. It has been suggested that LPS mediates renal tissue inflammation by activating TLR2 and TLR4‐related pathways [120, 121, 122]. Subsequent studies demonstrated that LPS can bind to TLR to promote the inflammatory cascade and cytokine expression, activating through the MyD88/NF‐κB pathway, up‐regulating the expression of tumor necrosis factor‐α(TNF‐α) and interleukin IL‐6, and down‐regulating the expression of COX‐2, iNOS, pro‐inflammatory cytokines, and nitric oxide [123, 124]. Repair of the LPS/TLR4/TRIF/NF‐κB axis can significantly up‐regulate mRNA expression of tight junction proteins Claudin‐1, Occludin, and ZO‐1, and reduce intestinal inflammation and oxidative stress damage [125]. LPS can also cause insulin resistance through TLR4 [126]. Binding of LPS to these cell receptors enhances the inflammatory response and may lead to damage and dysfunction of islet cells, affecting insulin secretion and action [127]. It may also cause inflammation through systemic circulation to the kidney, which becomes an important cause of DN. To sum up, LPS plays a complex role in DN, but the current research is not deep enough (Figure 2).

#### 2.4.5 Others

The metabolism of toxic products in gut microbiota is a complex process involving many steps and enzymes. Under normal circumstances, the human body can form a metabolic network of toxic products in gut microbiota [128]. However, when the intestinal barrier is damaged or the intestinal microbiome is changed, they will disrupt the stability and function of the intestinal tract in patients with DN, affecting the overall health of the host. Uremic Toxin is often called Protein‐Bound uremic toxin because it binds to proteins and is not easily excreted. Available evidence suggests that uremic toxins may increase due to increased abundance of *Enterobacteriaceae*, *Clostridiaceae*, *Pseudomonas*, and *Bacteroideaceae*, while decreased levels of *Lactobacillaceae*, *Bifidobacteriaceae*, and *Prevotellaceae* [48, 49]. Related research focuses on indoxyl sulfate (IS), which is produced by the fermentation of tryptophan into indole, which is then produced by endogenous oxidation and sulfonation [129]. IS is a ligand of the aromatic receptor (AHR), which plays a key role in the regulation of podocyte function [130]. Sustained activation of AHR can lead to podocyte damage and accelerate renal fibrosis [131]. In addition, IS can induce proinflammatory macrophage activation, oxidative stress, and mitochondrial autophagy to promote kidney injury [132, 133, 134]. The available evidence shows that the serum IS level of DN mice induced by streptozotocin (STZ) is 4 times higher than that of the control group [50]. But this is reversible. Hou et al. demonstrated that sulfotransferase 1A1 could be used as a target to inhibit the accumulation of IS in the kidney and alleviate UO‐induced renal fibrosis in mice [135].

H_2_S is produced by a variety of bacteria homologous to mammalian cystatin β‐synthase, cystatin γ‐lyase, and 3‐MST enzymes, as well as by sulfate‐reducing and desulphurizing bacteria [136]. According to Kundu et al., H_2_S treatment reversed MMP‐9‐induced renal remodeling in DN mice and increased the expression of CBS and CSE [137]. H_2_S down‐regulates the phosphorylation of p66Shc through sulfonation of Cys59 residues, thereby reducing the production of ROS [138]. Recent studies have shown that H_2_S recruits iNOS to produce NO, inhibits the expression of high glucose‐induced NADPH oxidase 4 (NOX4), and then inhibits ROS by down‐regulating adenosine monophosphate kinase‐activated protein kinase (AMPK) in renal cells [139].

In summary, uremic toxins and hydrogen sulfide in the gut have an important impact on DN, not only in the pathogenesis of the disease but possibly as part of treatment.

## 3 Treatment of DN by Gut Microbiota

### 3.1 Micro‐Ecological Preparations

#### 3.1.1 Probiotics

For such a complex mechanism of the disease, a multi‐faceted approach to treatment needs to be considered to combat these pathogenic mechanisms. Due to the operability of probiotics, they are now used in DN research. Koshida et al. showed that the progression of type 2 diabetes and DN is primarily influenced by the accumulation of indoxyl sulfate and sulfate‐to‐cresol, while the development of ESRD is influenced by these enteric‐borne uremic toxins [140]. Probiotics can compete with pathogens for limited replication sites, thereby excluding toxins, enhancing mucosal barrier function, and enhancing epithelial cell integrity [141]. In addition, the findings of Zhang et al. and Ghosh et al. suggested that sustained supplementation with a single or multiple probiotics can improve renal metabolic markers in patients with kidney disease, including reducing HBA1c, fasting blood glucose, and microproteinuria/creatinine ratio, and reducing adiponectin levels, which is conducive to improving insulin resistance, increasing ghrelin concentration, and improving kidney function [142, 143]. Amelia et al. demonstrated that the probiotic *Dadiah* activates Sirtuin‐1, reduces TNF‐α to lower inflammation levels, and protects the kidneys in patients with DN [144].

#### 3.1.2 Prebiotics

Prebiotics can be broken down and absorbed by beneficial bacteria in the intestines, thereby promoting their growth and reproduction, such as *Bifidobacterium* and *Lactobacillus* [145], improving intestinal immunity by regulating the intestinal microecological balance [146]. Meanwhile, prebiotics can promote the decomposition of proteins, fats, and carbohydrates by beneficial bacteria in the intestines, thereby improving the utilization rate of nutrients [147]. Although commonly used prebiotics include oligofructose and oligogalactose [148], our survey found that most literature is focused on the application of polysaccharides; plant and herbal extract polysaccharides can alleviate tubular epithelial inflammation, cell apoptosis, and oxidative stress, increase autophagy to treat DN [149, 150, 151, 152, 153]. Wu et al. believe that restoring the function of glomerular epithelial cells in high‐sugar‐damaged kidneys is also one of the effective treatment methods for DN [154].

#### 3.1.3 Synbiotics & Postbiotics

Both synbiotics and postbiotics are related to the gut microbiota and may play a significant role in the prevention and treatment of DN [155]. Synbiotics, a combination of probiotics and prebiotics, have a synergistic effect that can enhance the maintenance of intestinal health by probiotics [156]. Baroni et al. demonstrated that synbiotics supplementation led to significant improvements in glycosylated hemoglobin (HbA1c), fasting blood glucose (FPG), and insulin levels in diabetic patients [157]. In contrast, the study by Jayedi et al. showed only modest reductions in HbA1c and FPG, failing to provide sufficient evidence to support Synbiotics as a treatment for DN [158]. Postbiotics refer to bioactive metabolites produced by probiotics during their growth and metabolism, including SCFAs, vitamins, antioxidants, etc. [159]. Although recent literature suggests that postbiotics may offer therapeutic benefits for diabetes, diabetic retinopathy, and inflammatory diseases [160, 161, 162]. In animal experiments, Kim et al. demonstrated that the addition of *Lactiplantibacillus plantarum LRCC5314* reduced corticosterone levels, increased SCFAs production, and improved insulin sensitivity [163]. For all that, current studies do not provide sufficient intuitive evidence of their effectiveness in improving outcomes in patients with DN, such as intestinal symptoms, quality of life, renal toxin levels, or kidney function [158, 161, 162, 163, 164]. Further research is needed to explore the specific roles and effects of these substances in DN.

### 3.2 Fecal Microbiota Transplantation

The fecal microbiota transplantation (FMT) has garnered significant attention in the treatment of DN in recent years [165]. From the clinical perspective, Shang et al. utilized 16S rRNA sequencing to analyze the gut microbiome composition of DKD patients and subsequently validated the findings in DN mice, demonstrating that FMT therapy can mitigate DN by influencing the harmful pathogens [36]. In animal experiments, Chen et al. orally administered fecal bacterial extracellular vesicles (fBEVs) and demonstrated that the microbial round outer membrane vesicles induced tubulointerstitial inflammation and kidney injury by activating caspase‐11 [166]. Similarly, Bastos et al. have reported that they conducted FMT treatment on BTBR^ob/ob^ mice and arrived at similar conclusions, demonstrating that FMT treatment can ameliorate insulin resistance and maintain intestinal structural integrity in BTBR^ob/ob^ mice [167]. Additionally, the study by Lu et al. also provides evidence that FMT can effectively enhance podocyte insulin sensitivity and alleviate tubulointerstitial and glomerular damage [168]. Nonetheless, it is important to acknowledge that fecal microbiota transplantation (FMT) continues to encounter several challenges and issues. The standardization and regulation of FMT demand urgent attention, and the indications and contraindications require further elucidation. Moreover, the long‐term efficacy and safety of FMT necessitate additional research and evaluation [165, 169]. Despite these challenges, FMT is considered to hold significant promise for the treatment of DN.

### 3.3 Dietary Fiber

**FIGURE 3:**
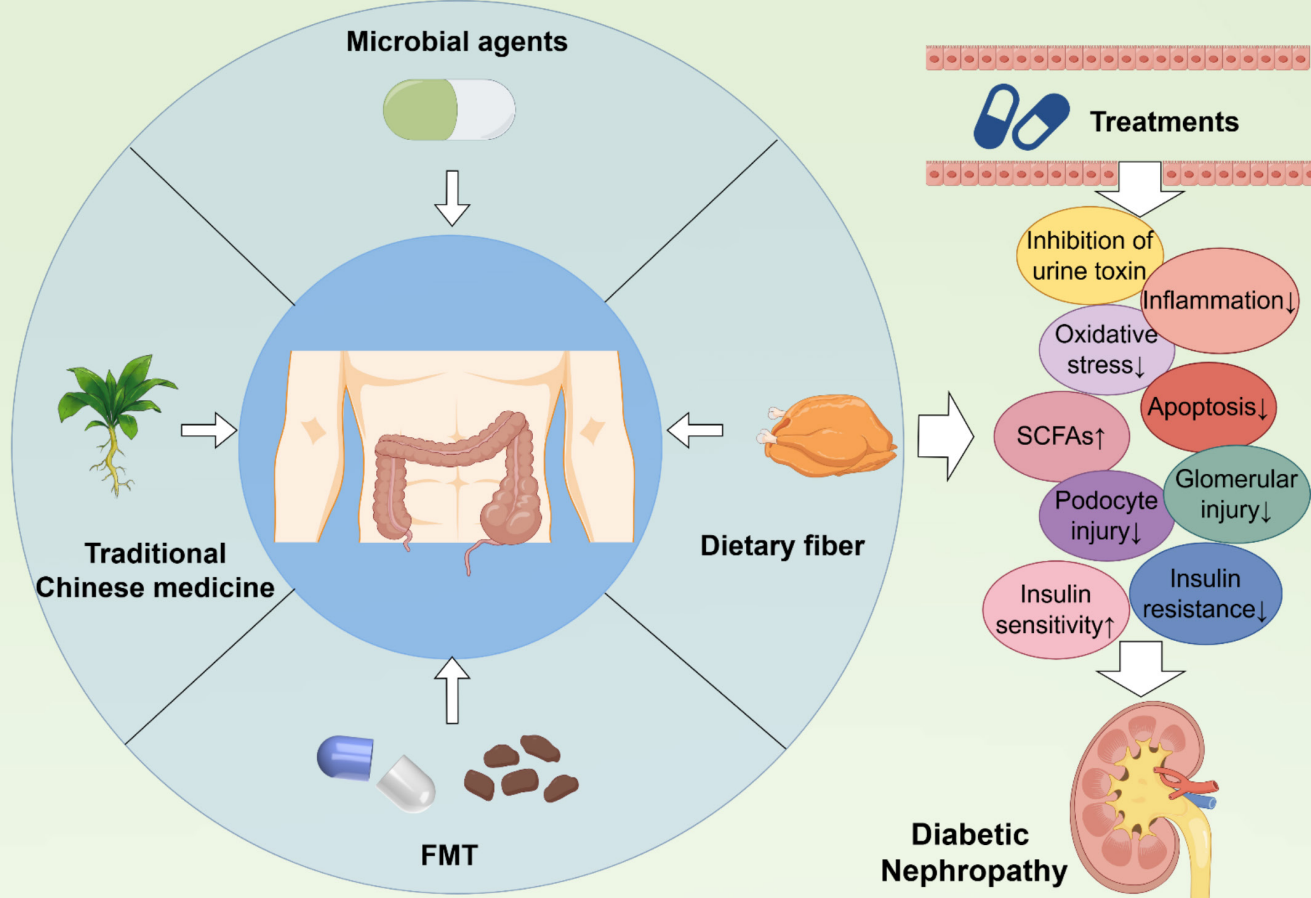
Current status of gut microbiota therapy for DN.

The role of dietary fiber supplementation in the treatment of DN has gradually attracted attention [91, 170, 171]. Dietary fiber refers to carbohydrate polymers that cannot be hydrolyzed by human digestive enzymes, and can be divided into two categories: soluble dietary fiber (SDF) and insoluble dietary fiber (IDF) [172]. Wu et al. found that dietary fiber supplemented with ingredients such as novel pectin can prolong the residence time of food in the gut and reduce the rate of glucose absorption, thus slowing the rise of blood sugar after meals [173]. In addition, dietary fiber can also increase insulin sensitivity and reduce insulin resistance [174]. Lin et al. found in their study that SDF extracted from Hazelnut can improve serum lipid parameters in rats on a high‐fat diet, and can also regulate intestinal SCFAs, significantly balance the abundance of *Lactobacillus*, *Roseburia*, and *Ruminococcaceae_UCG‐005* [175]. According to the conclusion of Xu et al., dietary fiber can reduce urinary protein excretion in patients with DN, reduce inflammatory response, and relieve the burden on the kidney [176]. Tanes et al. have demonstrated that dietary fiber plays a crucial role in maintaining the composition of gut microbiota, enhancing intestinal barrier function, and mitigating intestinal inflammation and oxidative stress, potentially through its modulation of both carbon‐based and nitrogen‐based metabolites [177]. Therefore, although the role of dietary fiber in DN has been confirmed to a certain extent, there are still many problems that need to be further explored (Figure 3).

### 3.4 Other Treatments

In addition to the aforementioned treatment methods, there are numerous novel extracts and methodologies utilizing gut microbiota to impact DN. Wang et al. utilized Paricalcitol (PAR), a vitamin D receptor (VDR) agonist, to demonstrate that VDR activation attenuates ferroptosis in DN proximal tubular epithelial cells (PTECs) through modulation of the Nrf2/HO‐1 signaling pathway [178]. This suggests that VDR plays a beneficial role by inhibiting ferroptosis, and future studies may use it as a target for drug therapy. Hirudin, extracted from leeches, is considered by Tian et al. to possess anti‐coagulation, anti‐fibrosis, anti‐scorch death, and anti‐inflammatory properties, and to have a significant protective effect on DN [179]. Similarly, Wu et al. believed that Marine sulfate polysaccharide (MSP) exhibits anticoagulation, regulation of glucose and lipid metabolism, and antioxidant effects in vivo and in vitro [154].

In addition to the gut microbiota, some researchers have also focused on the viruses in the gut. Rasmussen et al. demonstrated that transferring cecal virus communities from lean mice to high‐fat fed mice improved glucose tolerance and normalized blood glucose levels, likely due to the antagonistic relationship between phages and host bacteria [180]; further, Fan et al. noted that strong virus‐bacterial interactions in humans are disrupted in T2D and DN, and the enterovirus community is mainly composed of bacteriophages (phages) that target bacteria and play a crucial role in DN [181, 182]. In patients with DN, *Bacteroides phage*, *Anoxybacillus virus*, and *Brevibacillus phage* were deficient, while *Shigella phage* and *Xylella phage* were enriched [182]. The regulation of the gut virome may also be a means to treat DN in the future.

## 4 Conclusion and Prospects

Gut microbiota play an important role in the occurrence and development of diabetic nephropathy. The structure and composition of the gut microbiota were also altered in diabetic nephropathy patients. Disruption of the intestinal barrier causes bacteria to shift and toxic substances to enter the bloodstream. The inflammatory response and the production of SCFAs in the intestine are reduced, and DN is aggravated through the enterorenal axis. These changes may be related to the changes of gut microbiota metabolites, which further affect the health of the kidney. SCFAs, TMAO, BAs, and LPS, as major metabolites, are involved in regulating insulin and leptin secretion, insulin resistance, and cholesterol accumulation. Based on this, interventions targeting gut microbiota can be formulated, which may provide new ideas for the treatment of T2DM and DN. For example, the rational application of probiotics, prebiotics, and other micro‐ecological preparations, or exploring possible methods of FMT. It has certain clinical significance to help DN patients regulate the gut microbiota environment to alleviate the further deterioration of renal function.

Most current studies focus on metabolomic analysis of gut microbes, with the aim of finding evidence of a relationship between gut microbiota and DN [26, 44]. Some studies also reflect the changes and correlations in different stages of DN progression [21]. However, the limited sample size in clinical research has led to a stagnation of progress within individual research circles, hindering the advancement of precision medicine due to a lack of connectivity and collaboration among studies. In order to develop more accurate biomarkers that integrate kidney and gut microbiota, we are collecting kidney tissue and fecal tissue from DN patients, hoping to bridge the gap between them. 16 s rRNA V3 V4 variable region sequencing and Bulk RNA‐seq were used to analyze the transcriptional relationship between the microbiota and the disease and to explain the possible pathogenic bacteria groups. The aim is to explore the possibility of regulating the microbiota for the treatment of DN in the future, to provide new strategies and methods for the prevention and treatment of diabetic nephropathy and to bring better therapeutic effects for patients.

## Author Contributions

**Jinzhou Liu:** investigation, data curation, visualization, writing – original draft. **Min Guo:** project administration, funding acquisition, software, writing – review and editing. **Xiaobin Yuan:** conceptualization, methodology, resources. **Xiao Fan:** investigation. **Jin Wang:** funding acquisition, validation. **Xiangying Jiao:** funding acquisition, supervision.

## Disclosure

The authors have nothing to report.

## Conflicts of Interest

The authors declare no conflicts of interest.

## Supporting information


**Data S1.** Supporting Information.

## Data Availability

Data will be made available on request.
