# Supplementary material for: Gut Microbiota and Their Metabolites: The Hidden Driver of Diabetic Nephropathy? Unveiling Gut Microbe's Role in DN
Source: J Diabetes. 2025 Apr 6;17(4):e70068. doi: 10.1111/1753-0407.70068 (PMC11973130; doi:10.1111/1753-0407.70068)
Supplement: Supplementary file 1 — Data S1. Supporting Information. [file JDB-17-e70068-s001.docx]

**ETHICAL AND LEGAL DECLARATIONS**

**This review paper strictly adheres to the code of ethics and laws and regulations of academic research. This study is a review in nature and does not involve specific experimental or clinical studies, so it is suitable for any human or animal use and does not violate any individual rights. However, we continue to place a high value on the reliability and objectivity of our research, ensuring that the literature and data cited are from trusted sources and that appropriate attribution has been made in the text.**

**In terms of funding sources, we thank the following organizations for their support and funding:**

**1. 2023 Open fund scientific research project of Big Data Laboratory for Clinical Decision-making Research, Shanxi Medical University: Provided important financial support for the writing of this review, enabling us to comprehensively and systematically review the research progress in related fields.**

**2. Fund Program for the Scientific Activities of Selected Returned Overseas Professionals in Shanxi Province: The purpose of this review is to promote academic exchange and development in the field of diabetic nephropathy and gut microbiota.**

**3. The National Natural Science Foundation of China, as the director, provided financial and technical support for the completion of this review, which is conducive to promoting the transformation and application of scientific research results.**

**In terms of author contribution, we define the following division of labor:**

1. **Jingzhou Liu: As the main author of this review, she was responsible for the design of the overall framework, the selection and sorting of literature, and the writing and revision of the first draft.**
2. **Min Guo: I assisted in the collection and screening of a large number of literatures, and carefully proofread and supplemented the contents of the review.**
3. **Xiaobin Yuan: I participated in the writing of part of the review, and put forward valuable suggestions on the overall structure and logical relationship.**
4. **Xiao Fan: I reviewed the first draft of the review comprehensively, pointed out the existing problems and gave specific suggestions for modification.**
5. **Jin Wang: As a senior expert, he supervised and guided the research process in a comprehensive way, especially in the aspects of data interpretation and paper revision.**
6. **Xiangying Jiao: As a senior expert, he conducted the final review and check on the whole review to ensure the accuracy and authority of the content.**

**Our five authors jointly endorse the final version of this review and accept responsibility for the authenticity and completeness of its content.**
